# Improving hydrocarbon toxicity tolerance in poultry: role of genes and antioxidants

**DOI:** 10.3389/fgene.2023.1060138

**Published:** 2023-06-14

**Authors:** Vivian U. Oleforuh-Okoleh, Akeem B. Sikiru, Iyenemi I. Kakulu, Barineme B. Fakae, Uchechukwu E. Obianwuna, Ayoola J. Shoyombo, Adewale I. Adeolu, Ollor A. Ollor, Onyinyechi C. Emeka

**Affiliations:** ^1^ Department of Animal Science, Rivers State University, Port Harcourt, Rivers State, Nigeria; ^2^ Department of Animal Science, Federal University of Agriculture, Zuru, Kebbi State, Nigeria; ^3^ Department of Estate Management, Faculty of Environmental Sciences, Rivers State University, Port Harcourt, Nigeria; ^4^ Department of Animal and Environmental Biology, Rivers State University, Port Harcourt, Rivers State, Nigeria; ^5^ Insititute of Feed Research, Chinese Academy of Agricultural Sciences, Beijing, China; ^6^ Department of Animal Science, College of Agricultural Science, Landmark University, Omu-aran, Kwara State, Nigeria; ^7^ Department of Agriculture, Animal Science Programme, Alex-Ekwueme Federal University, Ikwo, Ebonyi, Nigeria; ^8^ Department of Medical Laboratory Science, Faculty of Science, Rivers State University, Port Harcourt, Rivers State, Nigeria

**Keywords:** antioxidants, epigenetic modulation, gene expression, hydrocarbon pollutants, oxidative stress, toxicity

## Abstract

Sustenance of smallholder poultry production as an alternative source of food security and income is imperative in communities exposed to hydrocarbon pollution. Exposure to hydrocarbon pollutants causes disruption of homeostasis, thereby compromising the genetic potential of the birds. Oxidative stress-mediated dysfunction of the cellular membrane is a contributing factor in the mechanism of hydrocarbon toxicity. Epidemiological studies show that tolerance to hydrocarbon exposure may be caused by the activation of genes that control disease defense pathways like aryl hydrocarbon receptor (AhR) and nuclear factor erythroid 2p45-related factor 2 (Nrf2). Disparity in the mechanism and level of tolerance to hydrocarbon fragments among species may exist and may result in variations in gene expression within individuals of the same species upon exposure. Genomic variability is critical for adaptation and serves as a survival mechanism in response to environmental pollutants. Understanding the interplay of diverse genetic mechanisms in relation to environmental influences is important for exploiting the differences in various genetic variants. Protection against pollutant-induced physiological responses using dietary antioxidants can mitigate homeostasis disruptions. Such intervention may initiate epigenetic modulation relevant to gene expression of hydrocarbon tolerance, enhancing productivity, and possibly future development of hydrocarbon-tolerant breeds.

## Introduction

Hydrocarbon pollution from oil spillage, natural gas flaring and organic pollutants is ubiquitous and of some fundamental health welfare concern in communities exposed to that pollution such as the Niger Delta region of Nigeria (Sam and Zabbey, 2018; Srivastava et al., 2019). The degradation of the environment by these pollutants destabilizes the ecosystem (Anejionu et al., 2015; Kponee et al., 2015; Croitoru et al., 2020), leading to the loss of biodiversity of organisms and low productivity of aquatic organisms, which serve as the major source of livelihood and animal protein supply to rural households (Izah, 2015; Osuagwu and Olaifa, 2018; Ansah et al., 2022). Diversification of income sources was recommended as a crucial strategy for achieving poverty reduction and economic stability in order to increase resilience and reduce the vulnerability of the rural poor to the effects of environmental deterioration (Yakubu, 2017). Poultry may be a good substitute due to its intrinsic qualities of having a short generational gap, high prolificacy, rapid turnover, and acceptance across all socio-cultural, economic, and religious strata (Oleforuh-Okoleh, 2010).

**FIGURE 1:**
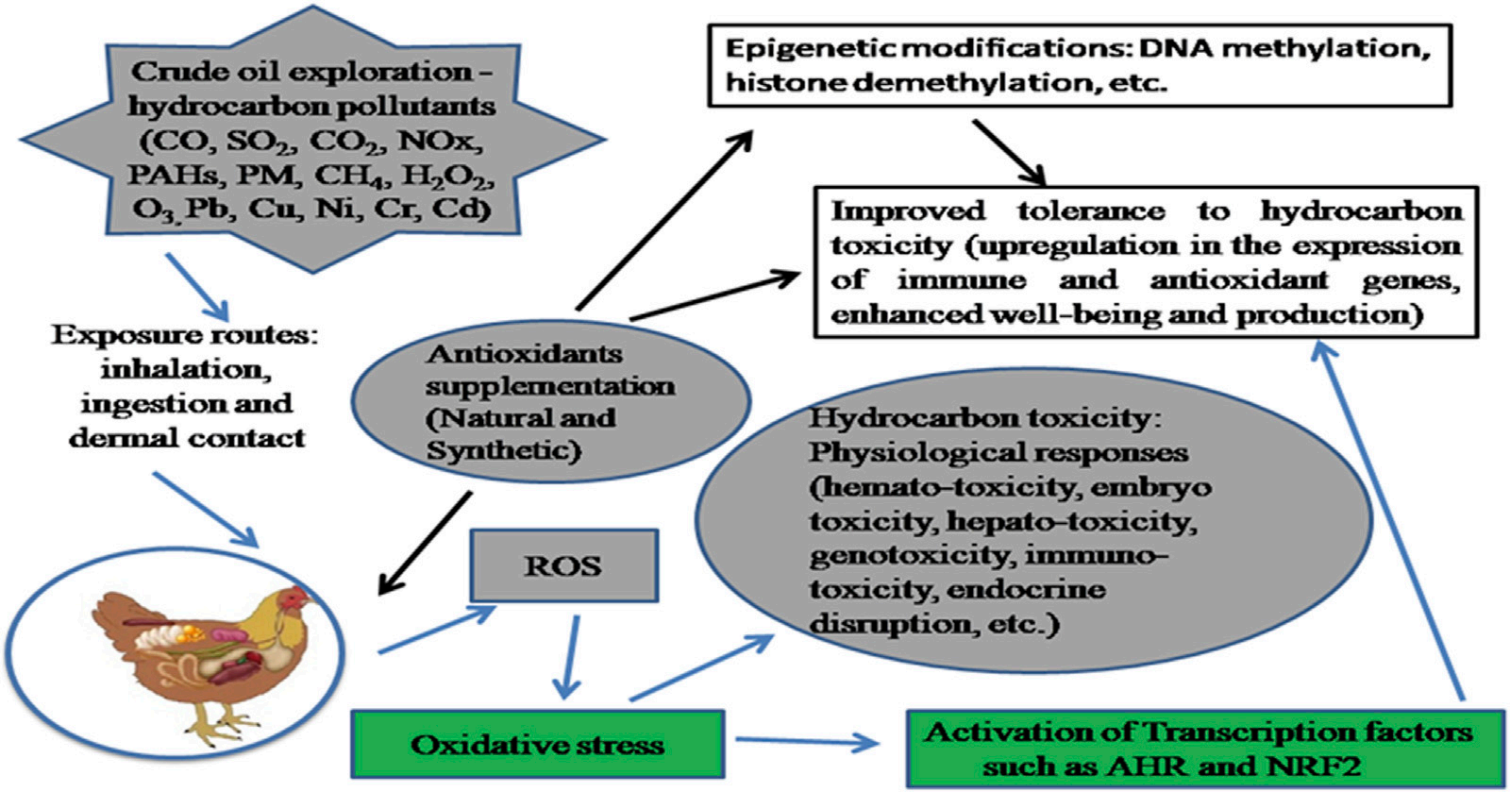
Consequence of hydrocarbon exposure and mitigation effect of antioxidant modulation. Crude oil exploration results in the release of hydrocarbon pollutants to the environment. Exposure to these pollutants via inhalation, dermal contact and/or ingestion results in interactions between different classes of pollutants, generation of reactive oxygen species and onset of oxidative stress conditions which are partly modulated by redox-sensitive signalling proteins and transcription factors. The mechanism of tolerance to hydrocarbon toxicity involves the activation of the aryl hydrocarbon receptor (AHR) and nuclear factor erythroid 2p45-related factor 2 (Nrf2) signalling pathways which regulates the expression of xenobiotic metabolism genes such as the cytochrome P450. To mitigate the impact of exposure on metabolic processes and enhance productivity, dietary interventions in regulating environmental perturbances could be beneficial.

Due to their toxic effects, environmental stresses such as those caused by hydrocarbon pollutants may prevent the full expression of the genetic potential of these birds. The severity of hydrocarbon toxicity is dependent on the exposure route, type of chemical compound, dosage, and duration of exposure. Hydrocarbon tolerance is regulated by biological (metabolic, genetic, and signal transduction) pathways whose expression upon exposure may create species-specific tolerance variances (Miller et al., 2018). Interactions between different classes of pollutants, the generation of reactive oxygen species (ROS), and the onset of oxidative stress conditions are partly modulated by changes in the levels and functions of redox-sensitive signaling proteins and transcription factors (Regoli and Giuliani, 2014). Modulation of the biological pathways using enzymatic and non-enzymatic molecules that can prevent metabolic dysfunctions caused by oxidative stress becomes expedient (Figure 1). There is evidence of effective dietary interventions using exogenous antioxidants to regulate the impact of environmental perturbances on organisms and hence gene expression with regards to tolerance (Hennig et al., 2007; Hoffman et al., 2017; Andreescu et al., 2018; Ideraabdullah and Zeisel, 2018).

Epidemiological studies indicate that oxidative stress modulates the organism’s epigenome and thus regulates gene expression (Malireddy et al., 2012; Hedman et al., 2016). Karchner et al. (2006) and Bianchini and Morrissey (2020) highlighted the fact that there are species-specific differences in the mechanism and level of tolerance to hydrocarbon fragments. This disparity could be observed between chickens raised in severely hydrocarbon-polluted environments and those raised in unaffected areas. The extent of vulnerability could be due to evolutionary capability being dependent on factors such as the genetic structure and strength of the selection pressure (Dutilleul et al., 2015). Modifications of the genetic structures, such as those associated with fecundity, survival, and morphology, are experiential (Nacci et al., 2002; Charmantier and Garant, 2005). Whitehead et al. (2017) asserted that the genetic architecture core to adaptive traits may interrelate to form the possibility of evolutionary rescue from pollution. Thus, genomic variability is critical for adaptation and serves as a survival mechanism in response to environmental pollutants (Oziolor and Matson, 2015).

Crucial to the successful establishment of smallholder poultry production in regions affected by hydrocarbon pollution is, therefore, an understanding of the impact of hydrocarbon pollutants on poultry with respect to gene-environment interaction as well as the mitigation effect of such an impact using exogenous antioxidants. This review highlights some physiological dysfunctions arising from exposure to hydrocarbon pollutants while noting some mechanisms of hydrocarbon toxicity and connecting dietary modulation to possible epigenetic changes towards improving tolerance to enhance productivity.

## Hydrocarbon pollution and physiological responses of poultry

Pollutants (both primary and secondary) from crude oil exploration activities like gaseous emissions from incomplete fossil fuel combustion and petrochemical industries, particulate matter (PM), polycyclic aromatic hydrocarbons (PAH), volatile organic compounds (VOC), and heavy metals (Pb, Cu, Ni, Cr, and Cd) pose adverse health challenges not only to humans but to other organisms within the ecosystem (Table 1). The interference of free radicals and reactive oxygen species (ROS) on the function of the cellular membrane as well as the enzymatic systems could possibly explain the mechanism of hydrocarbon toxicity. Though free radicals in the body system are involved in normal cellular functions and are essential in reduction-oxidation reactions and other physiological responses, maintenance of the balance between oxidation and anti-oxidation is critical (Bouayed and Bohn, 2010). Exposure of populations to hydrocarbon pollutants results in an imbalance between free radicals and the cellular antioxidant defense system, which culminates in various mutagen-induced deleterious effects. Overproduction of ROS causes them to behave as molecular sharks whose function is to damage molecules of the cell membrane, mitochondria, and nucleic acids, leading to oxidative stress (Aher et al., 2011). In other words, active oxygen species serve as mediators of the pollutants induced physiological responses by acting as precursors to various types of diseases, thereby affecting production (Ekweozor et al., 2002), fitness, and survivability (Cohen et al., 2017).

**TABLE 1:** Hydrocarbon pollutants and their effect on physiological responses of different organisms.

Hydrocarbon pollutant	Sample	Population	Physiological response	References
Particulate matter (PM)_2.5_	Lungs	Human	PAH-Coated with PM induced gene expression of CYPIAL, NQO1, GST-P, 1 and GST mu-3	Abbas et al. (2009)
Biomass fuel (BMF) smoke	Blood and Lungs	Human	Downregulation of Nuclear factor Keap1 experimentation in BMF users, activation of Nrf2 and upregulation of NQO1	Mondal et al. (2018)
Ozone	Lungs	Rats, mice, guinea pig	Elevated lung enzyme activities, alveolar duct fibrosis in rat and guinea pig	Dormans et al. (1999)
Polycyclic Aromatic Hydrocarbons (PAHs)	Liver	Double crested cormorants	Mutations in DNA microsatellites of birds closest to pollutionsites	King et al. (2021)
PAHs			Epigenetic modifications (changes in DNA methylation, histone modification and miRNA regulation) resulting in chronic diseases	Das and Ravi (2022)
Ozone	Lungs	Quail	Loss of cilia in bronchi and trachea, necrosis of air capillary epithelium, inflammatory response	Rombout et al. (1991)
SO_2_, NOx, PM _2.5_	Blood	Passerine birds	Decrease in red blood cell count, beta globulins and body weight, increase in ESR size and liver transaminase	Llacuna et al. (1996)
Air pollutants	Lungs, feathers	Sparrow	Retention of particulate matters in the lungs and accumulation of toxic metals in the feather as well as lower T-AOC, SOD, immunoglobulin concentrations	Li et al. (2021)
PM	Genes		Epigenetic alterations (DNA methylation)	Ferrari et al. (2019)
Benzo{a}pyrene (BaP)	Blood	Broilers	Evidence of hemato- and hepatoxicity due to BaP oxidative stress	Latif et al. (2010)

The accumulation of petroleum hydrocarbons in different tissues of exposed organisms has been reported (Bursian et al., 2017; Alzahrani and Rajendran, 2019). Increased stress levels, elevated detoxification efforts, and impairment of reproductive fitness were reported in birds following inhalation of pollutants (Sanderfoot and Holloway, 2017). A varied degree of biochemical and cellular responses linked to protein catabolism, bile acid metabolism, glucose homeostasis, and lipid peroxidation was observed in birds found in oil spill regions (King et al., 2014; Bianchini and Morrissey, 2018). Certain diseases such as lipid pneumonia, pulmonary dysfunction, decreases in hemocrit values, and immune-toxic effects were diagnosed in exposed birds (Szaro, 1991; Leighton, 1993; Trust et al., 1994). Al-Badri et al. (2019) found early and progressive stages of apoptosis in the hepatocytes of ducks exposed to various sources of air pollution. Pollutants substantially impacted osmoregulatory mechanisms, resulting in a decline in kidney function in birds exposed to pollutants (Amakiri et al., 2009; Dean et al., 2017). In the human population, there is a correlation between maternal exposure to air pollution and an increased risk of impaired lung development in the progeny (Saha et al., 2018). Pre- and post-hatching examinations of fertile eggs of *Larus marinus* (Lewis and Malecki, 1984) exposed to petroleum revealed developmental abnormalities of the embryo, morbidity, and high mortality in various avian species (Albers, 1978; Albers, 2006; Dubansky et al., 2018). Ekweozor et al. (2002) noted that hens exposed to crude oil had diminished growth, egg production, egg quality, and hatchability. Kubale et al. (2018) and Jiang et al. (2019) reported incidences of cardiovascular diseases such as atherosclerosis, right ventricular failure, and developmental cardiotoxicity in poultry.

## Genes’ function in hydrocarbon toxicity

Variations in single-nucleotide polymorphisms from xenobiotic response elements (like hydrocarbon pollutants) influence gene expression (Liu et al., 2018). Lodovici and Bigagli (2011) reported on the mechanisms and pathways by which this occurs. There is evidence that these pathways are stimulated by oxidants in a number of cell types and may be involved in the activation of transcription factors (Samet et al., 2002; Øvrevik et al., 2015). The resultant physiological dysfunctions (Låg et al., 2020) are evidenced by abnormal cell differentiation and transcriptional activation of xenobiotic-activated receptors such as the aryl hydrocarbon receptor (AHR), nuclear factor erythroid-derived 2 (Nrf2), and nuclear factor (NF)-kappa B-related genes (Shukla et al., 2000; Watzky et al., 2022). A redox balance in oxidant-antioxidant synthesis is critical to cell signaling mechanisms, which are crucial for regulation of different gene expressions, adaptation to stress, and ultimately homeostasis maintenance in the body (Malireddy et al., 2012; Hedman et al., 2016; Surai et al., 2019).

AhR-mediated signaling is known for its role as a sensor for environmental stimuli (Karchner et al., 2006; Bessede et al., 2014; Zhou, 2016); it regulates the expression of many genes in response to xenobiotics. It has been proposed as a signal transducer of hydrocarbon-induced oxidative stress and inflammation (Dietrich, 2016; Jang et al., 2019), involved in the regulation of biological responses to polycyclic aromatic hydrocarbons (Ma and Baldwin, 2000; Zhou, 2016). It also regulates angiogenesis, hematopoiesis, drug and lipid metabolism, cell motility, and immune modulation (Puga et al., 2002). The cellular mechanisms of the gene showed that when a ligand binds to AhR, it moves into the nucleus, where it forms heterodimers and triggers transcription by binding to xenobiotic response enzymes like cytochrome P450 monooxygenases, aldehyde dehydrogenases 3, glutathione-S-transferases, and NADPH/quinone oxidoreductases (Gomez et al., 2018). Reitzel et al. (2014) indicated that different populations of Atlantic killfish showed strong genetic structure at the AhR-related loci studied.

Furthermore, genes that protect the cells against damage from oxidative stress (cytoprotective genes) are induced by antioxidant response elements at the transcription level mediated by Nrf2 (Jaiswal, 2004). Expression of Nrf2 occurs mostly in tissues easily affected by external stimuli like the gastrointestinal tract, lungs, and skin, as well as those that function in detoxification (Speciale et al., 2011). It plays a key role in the regulation of antioxidant biomarkers such as superoxide dismutase, glutathione peroxidases, glutathione S-transferases, and catalase (Zazueta et al., 2022). Evidence from chromatin immune-precipitation indicates that Nrf2 regulates the expression of AhR by binding to an antioxidant response element region in the AhR promoter (Shin et al., 2007). For instance, Shukla et al. (2000) reported an increase in transcriptional activation of NF-keppa B-dependent (NF-κB) gene expression, which was inhibited in the presence of catalase in murine exposed to PM_2.5_ at non-cytotoxic concentrations. Inhalation and absorption of pollutants in different respiratory organ epithelial cells have been observed to induce oxidative stress (Liu et al., 2018), causing an increase in the translocation of some NF-κB to the nucleus and their increased binding to DNA, leading to the expression of genes associated with NF-κB such as interleukin 6 (IL-6) and tumor necrosis factor alpha (TNF-α) (Jiang et al., 2019). A study on genes associated with fat deposition in sanderling (*Calidris alba*) revealed that exposure to PAH downregulated the liver basic fatty acid binding protein 1 (Lbfabp) and hepatic lipase (Lipc) expression (Bianchini et al., 2021). There is evidence of inflammatory and immune diseases associated with respiratory disorders arising from the AhR-dependent disease pathway (Neavin et al., 2018).

## Antioxidants and hydrocarbon toxicity

Antioxidants are molecules that interact with reactive oxygen species to cease their oxidative reaction. They function by neutralizing free radicals (nitrogen, oxygen, and lipidic radicals) and protecting the body’s systems. Exposure to hydrocarbon pollutants induces oxidative stress, which stimulates injurious, inflammatory, adaptive, and reparative processes that overwhelm the antioxidant. The toxicity effect on the health and wellbeing of the organism arises from the level of genomic instabilities and alterations expressed as damage to biological molecules like proteins, lipids, and nucleic acids (Ren et al., 2017), resulting in oxidative stress, morbidity, mutation, and mortality. Oxidative stress is considered one of the critical pathways for the metabolism of hydrocarbon pollutant effects in the body (Tashakkor et al., 2011). The maintenance of the antioxidant status of exposed poultry is critical to halting cell damage and disrupting normal physiological processes. Antioxidants function in three ways to lessen the harm oxidative stress causes. These include enhancement of the expression of intracellular antioxidant enzymes, inhibition of the activity of ROS-generating enzymes, and direct reactions with the ROS (Lü et al., 2010; Poljšak and Fink, 2014).

The use of cellular antioxidants in prevention and protection against the metabolic dysfunctions can be achieved through enzymatic and non-enzymatic antioxidant defense systems (Bagyi et al., 2021) Consequently, dietary supplementation of exogenous antioxidants to compensate for the deficit in endogenous ones is imperative (Santos-Sánchez et al., 2019). Exogenous antioxidants rich in phenolic compounds have been proven to possess high antioxidant capacity owing to their redox properties, which enable them to adsorb and neutralize or minimize ROS, quench, and decompose peroxides (Osawa, 1994; Chen et al., 2020). Dietary antioxidants have been demonstrated to influence gene expression involving biochemical and pathological changes with respect to metabolic tissues, immune function, and disease risk factors induced by oxidative stress (Dincer and Yuksel, 2021; Dunisławska et al., 2022). The anti-oxidative effects of dietary antioxidants in a bid to compensate for the deficit in the endogenous system are achieved through various mechanisms and the interplay of signalling molecules, including the mediation of AhR, Nrf2, and CYP1A1 (Grishanova, 2022). Food polyphenolics showed AhR-based interactions at high concentrations (Amakura et al., 2008). Administration of a 6-shogaol-rich extract from ginger resulted in the induction of Nrf2 and Ho-1 regulated by mitogen-activated protein kinases (Bak et al., 2012). Numerous studies have shown potential benefits of antioxidant supplementation in the upregulation of transcription factor genes (Nrf2 antioxidant genes) and downregulation of inflammatory pathways triggered by oxidative stress (Table 2). Much as phytonutrients are capable of down- or up-modulating AhR signalling, the mechanism of their action on AhR and the Nrf2 system is not clear.

**TABLE 2:** Impact of antioxidant dietary modulation on different cell functions.

Dietary contents	Population	Type of intervention	Physiological response	References
Eucalyptus leaf polyphenol	Chickens	Dietary supplementation	Upregulation of antioxidant genes like HAO_2_, GGT_1_, GSTA4L, MGSTI.Inclusion of extract enhanced GSH–Px activity and reduced MDA in muscle tissues thereby improving antioxidant states of chickens	Li et al. (2020)
Tea extract granule	Chickens	Oral administration (drinking water)	Induction of oxidative stress via intramuscular injection of cyclophosphamide	Chi et al. (2020)
			Supplementation increased body weight and elevated the activity of SOD, CAT, GPx, with reduced MDA	
Vitamin C and Vitamin E	Chickens (Lveyang black-boned breeder rooster)	Dietary supplementation	Oxidative stress induced by subcutaneous injection of dexamethasone. Induced stress decreased SOD, IgM and mRNA expression of SOD and GSH-Px. Supplementation with Vitamin C and E had beneficial effect during early growth phase with increased body weight, improved antioxidant ability and immune performance in oxidative stressed roosters through upregulation of the expression of GSH–Px gene	Min et al. (2018)
Nettle (*Urtica dioica*)	Broiler chickens (Ross 380)	Dietary supplementation	Over expression of catalase, superoxide dismutase 1 in the lungs and liver of treated group. Attenuation of the right ventricular hypertrophy. Upregulation of hepatic and pulmonary antioxidant genes	Ahmadipour and Khajali (2019)
Vitamin C	Broiler chicken eggs (Arbor Acres)	Inovo injection	Improved growth performance traits, hatchability, total antioxidant capacity, immune status, and splenic expression of IL-4 and DNMT1, increased in expression of 1L-6, IFN-γ and TNF—α	Zhu et al. (2020)
Genistein	Breeder hens	Dietary supplementation	Maternal supplementation of genistein alters lipid metabolism in offspring through epigenetic modification resulting in improved antioxidant capacity	Lv et al. (2019)
			Upregulation in the expression of peroxisome proliferator-activated receptor (PPAR) genes, induced histone trimethylation and acetylation in chick liver	
Pterostilbene	broilers	Dietary supplementation	Attenuates diquat induced hepatic injury and oxidative stress of broilers via significant increase expression of Nrf2, heme oxygenase1, SOD and glutamate-cystein ligase catalytic subunit	Chen et al. (2020)
Folic acid	broiler	*In ovo* feeding at embryonic age 11 days	Increased hepatic folate content and expression of methlenetetrahydrofolate reductase and methionine synthase reductase. Increased plasma lysozyme activity and IgG and IgM concentray = tion, Histone methylation in IL-2 and IL-4 promoters. Immune function and epigenetic regulation of immune genes enhanced	Li et al. (2016)
Ginger extract	Rat	Oral administration	Exposure to lead via drinking water to induce oxidative-hepatic toxicity. Treatment with ginger extract resulted in upregulation of mRNA expression of antioxidant genes GST-α1, GPx1 and CAT in ginger extract supplemented group. GE had an antioxidant protective effects against lead acetate induced hepatotoxicity	Mohamed et al. (2016)
Ginger	Humans	Dietary supplementation	Increase in the expression of FoxP3 and PPAR-gamma genes in treated group and downregulation of the expression of T-bet and RORyt genes	Aryaeian et al. (2019)
Phytogenic premix (ginger, lemon balm, oregano and thyme)	Broiler chickens	Dietary supplementation	Supplementation of phytogenic premix upregulated the expression of cytoprotective genes (SOD1, GPx2, NQO1 AND HMOX1) and modulated the expression of Nrf2 and Keap1	Mountzouris et al. (2020)

Dietary modulation of genes encoding xenobiotic metabolizing enzymes to speed up the onset of disease tolerance, like those associated with hydrocarbon toxicity, would be beneficial even in poultry. Proteomic and transcriptomic analysis identified nine candidate genes and two candidate proteins that improved antioxidant status in chickens given eucalyptus leaf polyphenol extract (Li et al., 2020). Broiler chickens that were fed nettle (*Urtica dioica*) had a significantly upregulated expression of some antioxidant genes (SOD1 and CAT), which obviously prevented pulmonary hypertension (Ahmadipour and Khajali, 2019). The expression of CAT and SOD genes in the heart and lung of chickens reared in cold and high-altitude environments was increased when birds were fed *Securigera securidaca* (Ahmadipour, 2018). Curcumin and ginger were found to induce a protective effect by upregulating the activities of antioxidative enzymes, thereby modulating oxidative stress (Lin et al., 2019; Gao et al., 2022). A reduction in inflammation from oxidative stress was seen in mice administered a polyherbal mix of ginger, Chinese date, and wood ear mushroom (Nakyam et al., 2022).

## Gene-antioxidant modulation and hydrocarbon tolerance

During the selection process for economically important traits, genetic and environmental factors play a crucial role. Observed phenotypic characteristics, such as hydrocarbon tolerance in poultry, will be dependent on various mechanisms and pathways involving gene expressions pertinent to maintaining the integrity of cells in the face of pollutant-induced oxidative stress. Homeostatic misbalance due to oxidative stress during exposure to hydrocarbon pollutants may induce modifications in specific epigenetic markers. To this extent, the accessibility of genes to the cellular proteins that modulate gene transcription–how, when and where a gene is silenced or activated is required (Burton and Lillycrop, 2019).

Epigenetic changes are reversible mitotically stable modifications that do not necessarily change the DNA sequence but affect the way the sequence is transcribed (Ibeagha-Awemu and Ying, 2021; Dunislawska et al., 2022). There are therefore heritable alterations in gene expression that do not necessitate any change in the DNA sequence (Jirtle and Skinner, 2007; Guerrero-Bosagna and Skinner, 2012). Epigenetic processes influence the ability of an organism to adjust to prevailing environmental perturbance and include DNA methylation, histone acetylation, and non-coding ribonucleic acids (Ju et al., 2020; Corbett et al., 2021). These modifications are classical epigenetic mechanisms involved in packaging the chromatin structure, regulating DNA damage, and repressing gene expression, among other transcriptional activities (Bannister and Kouzarides, 2011; Hunt et al., 2013). The alterations that occur through DNA methylation inhibit gene expression by silencing the gene (Dhar et al., 2021); that due to histone acetylation can be in the form of post-transcriptional and post-transitional modifications (Wang and Ibeagha-Awemu, 2021); while the non-coding ribonucleic acid is essential for regulating cellular differentiation and organism development. Of the three mechanisms, DNA methylation causes differentiated cells to develop a more stable and long-lasting methylation pattern that regulates tissue-specific gene expression by recruiting proteins involved in gene repression or inhibition of the binding of transcription factor(s) to DNA (Moore et al., 2013; Dhar et al., 2021).

The use of dietary antioxidants has been shown to be an effective strategy involving epigenetic DNA methylation and cessation of oxidative stress. Nayak et al. (2016) emphasized the role of epigenetic mechanisms in stress regulation. Improving production, immune-competence, general health and wellbeing of poultry, through dietary intervention to assure tolerance level maintenance could involve epigenetic mechanisms. In addition, diets rich in folates, vitamins C, E, A, and D, as well as polyphenol metabolites found in phytobiotics, have been shown to suppress certain diseases via modulation of DNA methylation, histone modifications, and subsequent epigenetic regulation of gene expression (Malireddy et al., 2012). Ideraabdullah and Zeisel (2018) provide a straightforward illustration of how diet regulates genomic responses and potential physiological outcomes by establishing, recognizing, and responding to epigenetic markers. Hong and Gurjit (2012) assert that exposure to particulate matter can induce glutathione depletion in the methylation cycle, thereby promoting epigenetic changes, whereas Madrigano et al. (2012) established that PAHs form adducts inducing DNA methylation (hypo and hyper) of specific genes linked to physiological dysfunctions and contribute more to the occurrence and development of diseases (Cao et al., 2020). Hernández-Cruz et al. (2022) also reviewed cadium-induced epigenetic alterations and the use of antioxidant compounds to counteract Cd-induced epigenetic alterations.

## Future perspective

Evaluation of the genetic architecture and risk assessment of various chicken strains exposed to hydrocarbon contaminants is crucial for the development of intensive, commercial, and cost-effective smallholder poultry production in hydrocarbon-polluted communities. Studies on the regulation of target genes associated with environmental stress and immune-regulatory mechanisms related to hydrocarbon pollutants can provide a better understanding of how the phenotype plasticity of chickens exposed to hydrocarbon pollution can be utilized to increase productivity. Since the primary mechanism of hydrocarbon toxicity is oxidative stress, supplementing the diets of chickens exposed to pollutants with antioxidants is crucial, and the possibility of the supplement inducing epigenetic changes that promote hydrocarbon tolerance should be investigated. Based on the premise that expression patterns regulated by epigenetic processes, particularly DNA methylation, serve as a conduit for transmitting environmental information across generations via parental germ lines, such a change could be transgenerational (Weyrich et al., 2016). These studies may contribute to the identification of molecular markers for the creation of hydrocarbon-tolerant breeds.

## Data Availability

The original contributions presented in the study are included in the article/Supplementary Material, further inquiries can be directed to the corresponding authors.
